# Millennial scale feedbacks determine the shape and rapidity of glacial termination

**DOI:** 10.1038/s41467-021-22388-6

**Published:** 2021-04-15

**Authors:** Stephen Barker, Gregor Knorr

**Affiliations:** 1 School of Earth and Environmental Sciences, Cardiff University, Cardiff, UK; 2 Alfred Wegener Institute, Bremerhaven, Germany

**Keywords:** Palaeoceanography, Palaeoclimate

## Abstract

Within the Late Pleistocene, terminations describe the major transitions marking the end of glacial cycles. While it is established that abrupt shifts in the ocean/atmosphere system are a ubiquitous component of deglaciation, significant uncertainties remain concerning their specific role and the likelihood that terminations may be interrupted by large-amplitude abrupt oscillations. In this perspective we address these uncertainties in the light of recent developments in the understanding of glacial terminations as the ultimate interaction between millennial and orbital timescale variability. Innovations in numerical climate simulation and new geologic records allow us to highlight new avenues of research and identify key remaining uncertainties such as sea-level variability.

## Introduction

**Fig. 1:**
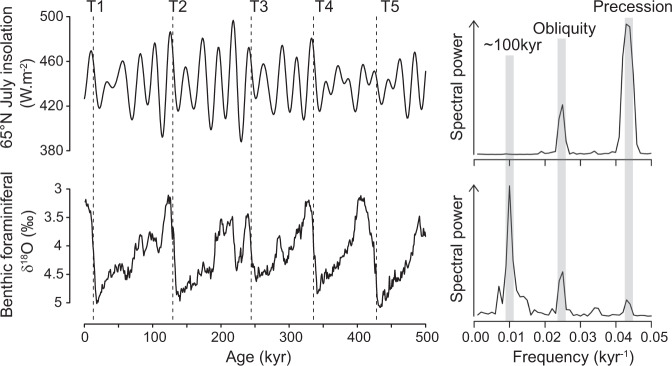
The 100 kyr problem. The geologic record of benthic foraminiferal δ^18^O^65^ contains significant spectral power in the eccentricity band (as well as those of precession and obliquity) while the external insolation forcing at this period (~100 kyr) is negligible. Late Pleistocene glacial cycles are ‘terminated’ by relatively short deglacial periods, giving rise to a characteristic saw-tooth character. T1–T5 represent Terminations 1 to 5.

In a seminal paper published in 1976 Hays, Imbrie and Shackleton^1^ provided the first convincing geologic evidence in support of Milankovitch theory^2^, which posits a direct link between Earth’s climate and changes in the geometry of our orbit around the Sun. One of the most remarkable observations made by Hays et al. was that the dominant period of climate variability over the last half million years or so (the glacial cycles of the Late Pleistocene) is close to 100 kyr (Fig. 1), even though the forcing at this period (changes in the circularity of Earth’s orbit, eccentricity) is negligible when compared to that related to obliquity (tilt of the rotational axis) or precession (timing of solstice relative to orbital position; Fig. 1). Whether glacial cycles are driven ultimately by changes in eccentricity or more likely by some combination of obliquity and precession^3–5^ (which itself is dependent on eccentricity) the question remains as to why glacial terminations should occur every ~100 kyr and not more frequently as they did during the early Pleistocene^6^. A clue to this question lies in the asymmetric nature of records of benthic foraminiferal δ^18^O (a crude proxy for continental ice volume; Fig. 1), which suggest a stepwise build-up of ice sheets over a period of ~90 kyr, followed by a shorter interval (~10 kyr) of relatively fast ice sheet decay during termination^7^.

Accordingly, most models of glacial-interglacial (G-IG) variability have called on the long-term build-up of continental ice sheets, and their propensity to collapse beyond some critical threshold, in order to explain the long-lived nature of late Pleistocene glacial cycles e.g. refs. ^3,4,8–12^. By this reasoning, only once ice sheets have exceeded some critical size, do they become susceptible to a potentially modest (external) forcing to which they respond in a non-linear way (Box 1). Indeed, it has long been appreciated that glacial terminations must involve non-linearities within the climate system in order to amplify the rather modest forcing from insolation^1,7,13^.

The specific suggestion that abrupt reorganisations of the ocean-atmosphere system might play an active role in the mechanism of termination was introduced by Mix et al.^14^ (although Ewing and Donn^15^ had predicted the importance of ocean circulation on the waxing and waning of continental ice sheets several decades earlier) and augmented to include a link to atmospheric CO_2_ by Broecker and Denton^16^. The most recent incarnation of this idea was summarised by Denton et al.^17^ as a sequence of events initiated by the inflow of freshwater to the surface North Atlantic (generated by summer melting of continental ice sheets) suppressing ocean circulation in the Atlantic and ultimately causing the release of CO_2_ to the atmosphere. At some critical point, the rise in CO_2_ would pass a threshold beyond which interglacial conditions could be stabilized and maintained^17–19^.

A number of recent studies provide evidence to support Broecker and Denton’s^16^ original proposition, with several independent lines of proxy evidence emphasising the ubiquitous association between glacial termination and millennial-scale oscillations in ocean circulation, in particular the Atlantic Meridional Overturning Circulation, AMOC^20–27^. Moreover, ice-core measurements and quantitative carbon cycle models support an active role for abrupt shifts in ocean circulation within the mechanism of glacial termination. For example atmospheric CO_2_ measurements in Antarctic ice cores combined with proxy reconstructions in marine sediments suggest that CO_2_ rises on a millennial timescale whenever Atlantic Ocean circulation is in a substantially weakened state^25,28–30^ with additional century-scale increases (at least occasionally) associated with abrupt transitions between weak and strong modes of ocean circulation^23,31,32^. Modelling studies further suggest that such changes in CO_2_ may be associated mechanistically with the corresponding variations in ocean circulation, either directly or indirectly through e.g. biological activity^33–39^.

However, while the simple model described by Denton et al.^17^ provides an appealing explanation for the magnification of insolation forcing related to deglaciation it leaves open several questions related to the interplay between millennial and orbital-timescale changes and the anatomy of terminations themselves, for example:

- Is the temporal correspondence between abrupt shifts in ocean circulation and glacial termination merely coincidence or are there systematic interactions between variations on these timescales that necessarily come into play during deglaciation?
- How do we explain the occurrence of terminal interruptions like the Bølling-Allerød/Younger Dryas (B/A-YD) oscillation during Termination 1 (T1) while other terminations proceed apparently without such interruption?
- Why do some terminations end with such high levels of atmospheric CO_2_ while others (notably the most recent) do not?

Below we address each of these questions in turn, highlighting new results and outstanding uncertainties requiring future research focus. We begin by investigating the occurrence of millennial-scale climate variability with respect to orbital-timescale changes in ice volume and atmospheric CO_2_.

Box 1 Glossary of key terms **Linear system** (Panel a): The equilibrated system response will be directly proportional to a change in the control parameter (e.g. freshwater forcing, atmospheric CO_2_, ice volume)

**Non-linear system** (Panel b): The equilibrated system response includes non-linear behaviour i.e. the response to a finite change in the control parameter depends on the location within the parameter space.

**Hysteresis** (Panel c): The response to a finite change in the control parameter depends on the location within the parameter space and different solutions can coexist (e.g. bistability) for a single value of the control parameter. Hence depending on the starting point different states can be attained for the same forcing. In this case a transient change in the control parameter, or e.g. a one-time input of freshwater, can have a lasting impact.

**Stability** (Panel c): A system may undergo changes in structural stability within the parameter space and alter e.g. from monostability to bistability (e.g. if hysteresis is present). In the case of monostability the system recovers to the initial state after a transient perturbation, while in the bistable phase space a transient change in forcing can have a lasting impact.

**Sensitivity** (Panels a, b): Increasing sensitivity will produce a stronger system response to a given change in the control parameter. In the case of non-linear systems and those with hysteresis the sensitivity to a given perturbation is dependent on the location within the phase space i.e. non-linearities and changes in stability behaviour can produce a stronger or weaker system response to a given perturbation.

**Window of opportunity** (Panel d): Conditions, which give rise to more frequent and larger amplitude millennial-scale climate variability. Specifically, the window of opportunity arises when both ice volume and atmospheric CO_2_ are at levels intermediate between their full glacial and full interglacial values. By definition, climate moves through the window of opportunity during glacial termination.

**Sweet spot** (Panel g): A maximum in the activity of millennial-scale climate variability within the window of opportunity.

**Moveable window of bistability** (Panels e, f): The stability properties of a system with respect to one parameter (e.g. atmospheric CO_2_) can vary as function of another parameter (e.g. ice volume). This can shift the region of bimodality in systems with hysteresis.
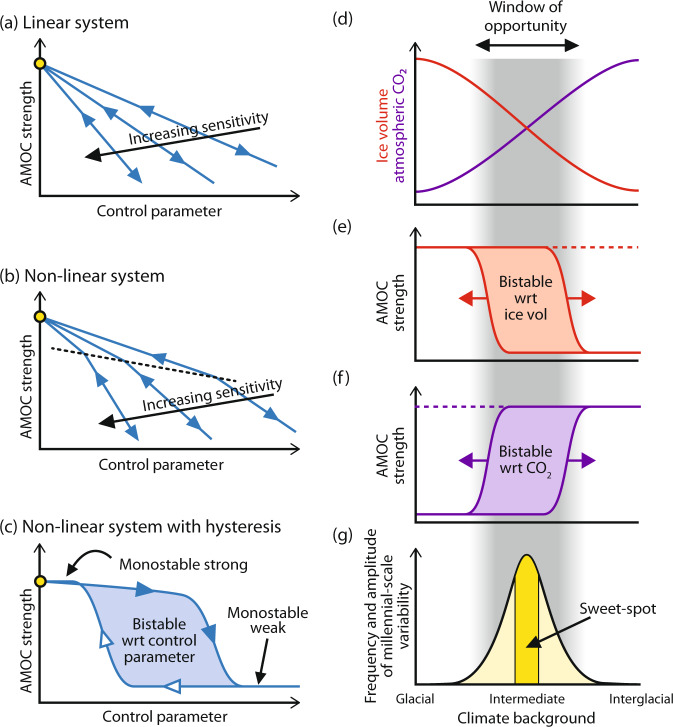

## Feedbacks between millennial and orbital-timescale variations

McManus et al.^40^ first described the pervasive nature of millennial-scale climate variability (Box 2) over the past 500 kyr and suggested that such variability was most pronounced when continental ice volume lay somewhere between full interglacial and extreme glacial values (Box 1); millennial activity was somehow dampened when ice sheets were relatively small (similar to modern) or particularly large. Since then, numerous studies have confirmed that millennial-scale climate variability experiences its greatest amplitude within intermediate climate states and transitions between states (notably terminations)^25,27,41–44^.

While geological observations suggest that there exist critical boundary conditions between which millennial-scale activity is enhanced, climate models can help us to identify which climatic parameters are most critical. Recent modelling studies have made some headway into this problem; Zhang et al.^45^ suggested that the large size of continental ice sheets could indeed be responsible for the (mono) stability of AMOC during full glacial periods, while high (interglacial) concentrations of atmospheric CO_2_ seem to be responsible for an equivalent stability during interglacial periods, a suggestion also supported by more recent studies^46,47^. When both parameters occupy the space between full glacial and interglacial conditions a ‘window of opportunity’ arises (Box 1) within which the AMOC exists in a bistable regime and may therefore be expected to experience more frequent and or pronounced variability. Abrupt variations can also occur within full glacial or interglacial conditions when we expect the AMOC to be monostable e.g. Heinrich Event 2 during the LGM or the 8.2ka event in the Early Holocene, but these are most likely transient perturbations forced by distinct events such as freshwater floods e.g. ref. ^48^ (Box 1).

Thus, millennial-scale variability seems to be dependent on orbital-timescale changes. Indeed, previous studies implied and demonstrated that gradual global warming can directly trigger abrupt transitions in the AMOC^49–52^, and it has now been shown that both ice sheet height and atmospheric CO_2_ can influence the strength of AMOC e.g. refs. ^46,53–56^ and the occurrence of abrupt changes^45,47,57–59^. Moreover, it is thought that abrupt variations in ocean circulation can have a reciprocal influence on CO_2_ (through resultant biophysicochemical changes^33–38^) and possibly sea level (although the actual relationship between variations in AMOC and ice volume remains controversial^60,61^), highlighting the possibility that millennial-scale variability may be considered both slave and master to orbital-timescale changes, an idea we develop below.

**Fig. 2:**
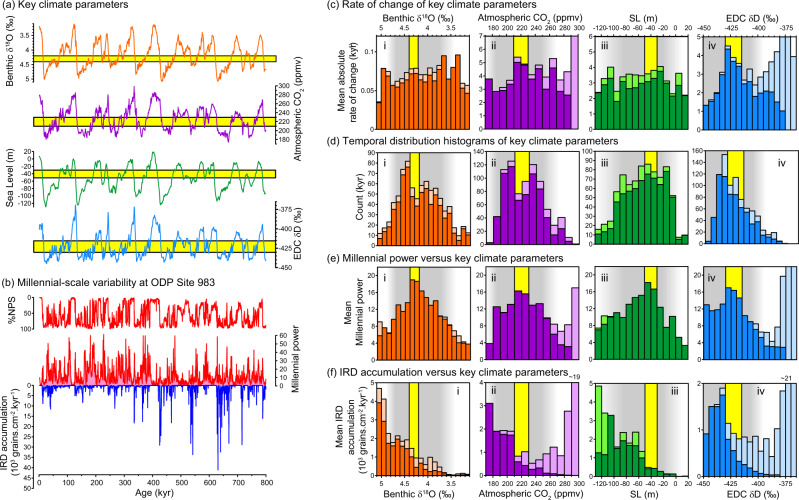
The sweet spot of millennial variability. **a** Records of benthic δ^18^O^65^, atmospheric CO_2_^97^, sea level^93^ and Antarctic δD^98^. Yellow bars are same as for **c**–**f**. **b** Records from ODP Site 983^25^: %NPS (a proxy for sea surface temperature), millennial power (Hilbert transform of the 0.5–7 kyr Taner bandpass filtered %NPS record) and Ice Rafted Debris (IRD) accumulation. **c** Binned mean absolute rates of change for the records in **a**. **d** Distribution histograms of the records in **a**. **e** Mean millennial power for the same bins utilized in **c**, **d**. **f** Mean IRD accumulation rates for the same bins utilized in **c**, **d**. In each panel fainter colours represent deglacial intervals (terminations) while bolder colours represent all other times. Yellow bars highlight the respective peaks in millennial power in **e** (the sweet spot as described in the text and Box 1) and grey shaded regions highlight the broader windows of opportunity.

Records of changing surface ocean conditions in the North Atlantic can be used to illustrate the interplay between millennial and orbital-timescale variability over the past 800 kyr (Fig. 2). Based on proxy evidence from the last glacial period^22,30^ we use variations in North Atlantic SST to infer changes in the AMOC. We quantify millennial power using a Hilbert transform (amplitude envelope) of the 0.5–7 kyr bandpass filtered %NPS (percentage of the polar affiliated foraminifera, *Neogloboquadrina pachyderma*, within the total assemblage) record from ODP Site 983^25^ (Fig. 2b). As expected, we observe enhanced power in the millennial-band associated with intermediate climate conditions (the window of opportunity, constrained here using benthic δ^18^O, atmospheric CO_2_ and relative sea level; Fig. 2e).

Less expected is the observation that the peak in millennial power (the sweet spot; Box 1) is associated with a local minimum in the distribution histograms of both benthic δ^18^O and atmospheric CO_2_ (Fig. 2d; the bimodal nature of CO_2_ variability was noted previously^62^). We suggest that these minima are manifestations of the reciprocal influence of millennial activity on the boundary parameters themselves. Specifically we propose that the feedbacks between abrupt changes in the AMOC and the global parameters of benthic δ^18^O and CO_2_ are sufficiently strong when millennial variability is at its maximum that the climate system is effectively forced away from the sweet spot and towards a more stable climate regime. If this is case then the occurrence and active role of abrupt climate shifts during glacial termination (and inception for that matter) is no mere coincidence, but rather represents the necessary and ubiquitous interaction between these two distinct modes of climate variability.

**Fig. 3:**
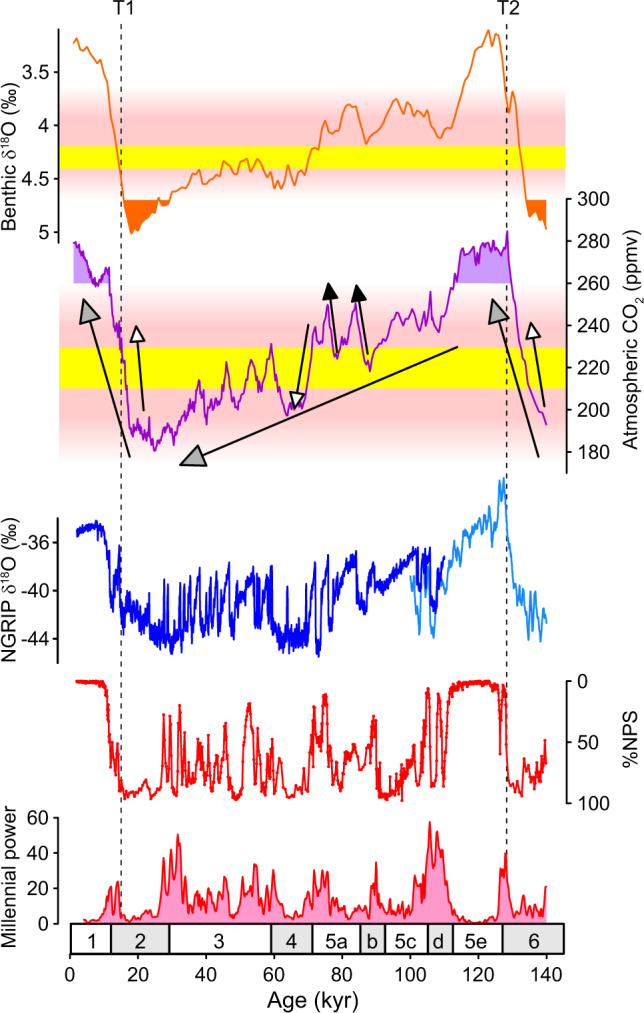
Feedbacks between millennial and orbital-timescale variability. From top to bottom: Benthic δ^18^O^65^ (filled regions indicate monostable AMOC as a function of maximum ice volume); Atmospheric CO_2_^97^ (filled regions indicate monostable AMOC as a function of high CO_2_); Greenland ice-core δ^18^O^99^ with synthetic record^27^ beyond 110ka; %NPS (temperature proxy) from ODP Site 983; Millennial activity at Site 983 (see text). Pink and yellow shaded boxes represent the window of opportunity and sweet spot respectively (see Fig. 2). Grey headed arrows represent orbital-timescale trends in atmospheric CO_2_; Black (white) headed arrows represent examples of negative (positive) feedback of millennial-scale variability on orbital changes. Feedbacks are strongest within the sweet spot. Dashed vertical lines indicate timing of AMOC recovery associated with glacial terminations T1 and T2. Lower numbers are Marine Isotope Stages.

For example, atmospheric CO_2_ experienced a long-term decrease from the last interglacial period (Marine Isotope Stage, MIS 5e) towards the LGM (MIS 2; grey headed arrow in Fig. 3), taking it from high concentrations (during which the AMOC was monostable strong and millennial activity was weak) to within the window of opportunity (during MIS 5d) and eventually the sweet spot (around the beginning of MIS 5b). According to our hypothesis at that point the feedbacks between millennial and orbital-timescale changes became more powerful and CO_2_ was forced upwards and away from the sweet spot (a negative feedback; black headed arrow in Fig. 3). However, CO_2_ continued its long-term decrease until MIS 3, during which it varied about an intermediate value of ~220 ppmv (within the sweet spot as indicated by our analysis; Fig. 2e ii). Indeed, paleo-reconstructions confirm (or at least suggest) that large and abrupt fluctuations in ocean circulation occurred repeatedly within MIS 3^22,30^ and these are thought to have strongly influenced atmospheric CO_2_, causing it to rise when circulation was particularly weak (e.g. during Heinrich events) and vice versa^25,36^. Thus, the effect of accentuated millennial-scale activity at that time was to continually drive CO_2_ in one direction or another depending on the mode of ocean circulation (i.e. feedbacks could be both negative and positive). On the other hand, the resulting changes in CO_2_ may themselves have reversed the oceanic conditions that produced them in the first place (e.g. ref. ^45^) thus forcing CO_2_ to remain within range of the sweet spot throughout MIS 3. Note that we are not suggesting that all D-O events were caused by changes in CO_2_ (or vice versa) and while this may have been the case for some of the larger events, we believe that high frequency D-O variability represents the inherent tendency of the AMOC to oscillate from one mode to another as an inevitable consequence of an intermediate climate background^63^.

**Fig. 4:**
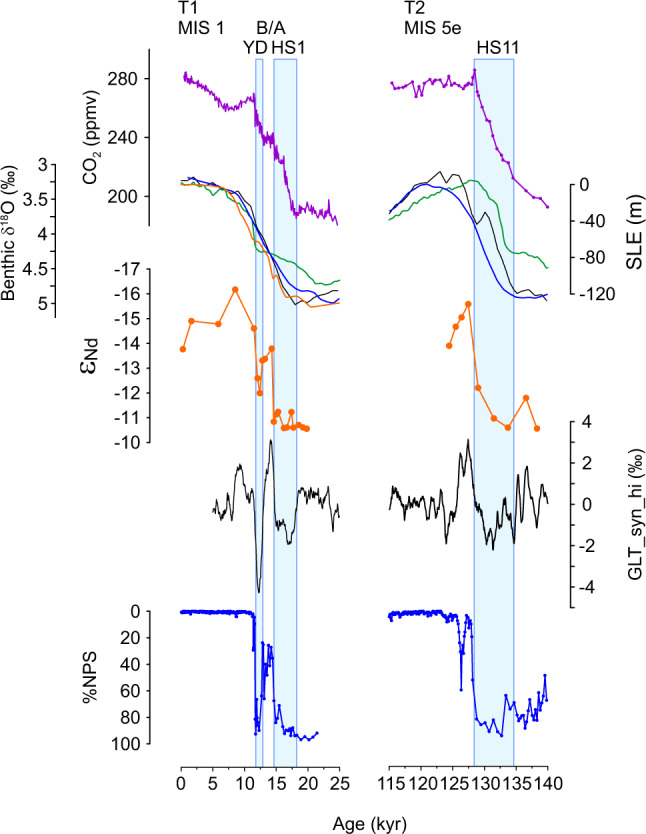
The last two terminations. From top to bottom: Deglacial timeseries of atmospheric CO_2_^31,97^, benthic δ^18^O (black curve^65^) and sea level reconstructions (orange^100^; green^92^; blue^93^), NW Atlantic sedimentary εNd^21,23^ (sensitive to ocean circulation change), GL_T__syn_hi^27^ (a low order approximation of AMOC strength^25^) and %NPS (a temperature proxy) from the NE Atlantic^68^. Blue boxes are cold (weak AMOC) intervals. T1, T2 are Terminations 1 and 2; MIS 1, MIS 5e are Marine Isotope Stages; B/A Bølling/Allerød, YD Younger Dryas, HS1, HS11 are Heinrich Stadials, within which Heinrich events, HE1 and HE11 occurred. SLE is Sea Level Equivalent.

CO_2_ declined further throughout MIS 3 and eventually ice sheets attained their maximum configuration during the LGM (and equivalently MIS 6), inducing a period of reduced millennial-scale activity associated with a monostable strong AMOC. Subsequently, during glacial termination, when climate again passed through the sweet spot, the effect of accentuated millennial-scale activity was to help push CO_2_ straight through the window of opportunity (a positive feedback; white headed arrow in Fig. 3), at least in the case of T2. Early recovery of the AMOC associated with the B/A during T1 (Fig. 4) slowed the rise of CO_2_ (a negative feedback) but the inevitable return to a weak mode of circulation during the YD (see sub-section: ‘solutions in the time domain’) enabled CO_2_ to increase beyond the window of opportunity with the onset of interglacial conditions, eventually returning to a monostable strong mode of AMOC during the Holocene (and MIS 5e). Notably, the occurrence of high amplitude millennial-scale variability associated with high (interglacial-like) CO_2_ is a peculiar feature of deglacial periods (Fig. 2e ii), which we ascribe to the influence of abrupt change on CO_2_ as opposed to the influence of CO_2_ on the occurrence of abrupt change (see also penultimate section).

As stated earlier, we are using variations in North Atlantic SST recorded at a single core site to infer large-scale changes in the AMOC and we admit that use of alternative records might give different results. For example, the coincidence of our observed sweet spot with minima in the histograms of CO_2_ and benthic δ^18^O could be mere coincidence and needs to be confirmed using alternative proxies and locations. Furthermore, the minima themselves are not particularly prominent, and could be a result of controlling factors other than millennial-scale AMOC variability. On the other hand, we observe a maximum in the absolute rate of change of Antarctic temperature (also proposed to reflect changes in the AMOC^27,64^) aligned with the maximum in millennial power at Site 983 (Fig. 2c iv), which also implies its coincidence with the local minima in CO_2_ and δ^18^O. This supports our contention that millennial-scale variability at Site 983 reflects variations in the wider climate system. We also note that the absolute rate of change of CO_2_ itself reaches a maximum (Fig. 2c ii) in line with the minimum in its histogram (Fig. 2d ii) and the sweet spot of millennial activity i.e. CO_2_ changes fastest when millennial activity is greatest, which we contend results in the local minimum in the histogram of CO_2_.

We also observe a peak in millennial power associated with intermediate ice volumes (−40 m Sea Level Equivalent, SLE; Fig. 2e iii). However, we do not find a corresponding minimum in the histogram of sea level. Because benthic foraminiferal δ^18^O (in this case the stacked record of ref. ^65^) represents a convolution of ice volume and deep ocean temperature this could mean that the local minimum in the histogram of benthic δ^18^O reflects the existence of two distinct modes in deep ocean temperature, which could correspond to the two equivalent modes in atmospheric CO_2_ (but note that such bimodality is not observed in Antarctic temperature; Fig. 2d iv), rather than ice volume alone. Alternatively, it could reflect the very large envelope of uncertainty associated with current sea-level reconstructions i.e. perhaps a local minimum in ice volume is obscured by inaccurate sea-level reconstructions. We return to this issue in the final section.

Finally, our analysis can inform us about the interactions between calving ice sheets around the NE Atlantic and millennial-scale climate variability. There has been some debate over whether or not iceberg calving events cause or amplify abrupt changes in ocean circulation (which can be inferred from changes in high northern latitude sea surface temperature)^66–68^. In a previous study we suggested that icebergs arrived too late to trigger cooling across the NE Atlantic. Here we use an extension of that dataset to show that the peak in millennial variability in SST at this site is misaligned with the peak in ice rafted debris accumulation, which we assume provides a first order indication of iceberg calving (e.g. compare Fig. 2e, f). In fact, with respect to all three parameters (benthic δ^18^O, CO_2_ and sea level) we note that ice rafting increases away from the sweet spot of millennial activity, reaching its maximum within the glacial mode of CO_2_ and δ^18^O and when ice volume exceeds −50 m SLE. While this observation does not negate the potentially significant influence of icebergs on SST (and ocean circulation), the fact that millennial variability in SST reaches its maximum power (maximum amplitude in the 0.5–7 kyr band) when ice rafting is still relatively subdued suggests that iceberg calving is not the main influence on millennial-timescale climate variability.

Box 2 Millennial-scale variability and abrupt climate change Aside from glacial cycles, Earth’s climate also varies strongly on millennial timescales in the form of Dansgaard-Oeschger (D-O) events^99^ (repeated oscillations between cold stadial and warm interstadial conditions across the wider North Atlantic region) and Heinrich events (HE; massive armadas of icebergs released across the North Atlantic^67^) associated with extended cold periods known as Heinrich stadials, HS (Box Fig. 2). The amplitude of D-O variability appears to depend on background climate state^25,27,40^, with highest amplitudes attained during intermediate states such as Marine Isotope Stage (MIS) 3 and much more subdued behaviour during interglacial (e.g. the Holocene) and full glacial periods (e.g. the LGM; see main text).

Transitions between stadial and interstadial states can occur very rapidly (within decades) and are thought to involve abrupt changes in the state of Atlantic Meridional Overturning Circulation (AMOC)^20,21,30^. Of particular relevance to this perspective are the abrupt climate shifts associated with glacial termination (e.g. the HS1-B/A-YD triptych during T1; Box Fig. 2). Significant weakening of the AMOC is thought to have accompanied both the HS1 and YD cold events^20,21^ while the B/A experienced a pronounced strengthening of AMOC. The release of CO_2_ and global warming associated with terminal stadial events such as HS1 and the YD is thought to accelerate the transition to an interglacial state^18,19^, providing a positive feedback on deglacial climate change (Fig. 4).

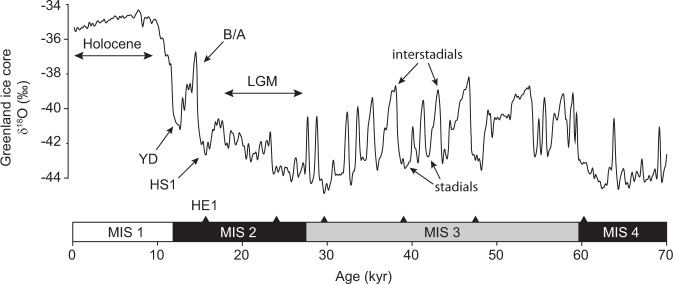

**Box Figure 2** Millennial-scale climate variability as documented by Greenland ice-core δ^18^O^99^ (a 1st order proxy for local temperature) reveals abrupt fluctuations between cold (stadial) and warmer (interstadial) conditions. YD Younger Dryas, B/A Bølling-Allerød, HS1 Heinrich Stadial 1, LGM Last Glacial Maximum. Black triangles represent Heinrich events (e.g. HE1).

## The Bølling-Allerød/Younger Dryas oscillation

The abrupt climatic oscillation represented by the HS1-B/A-YD triptych during Termination 1 (Box 2; Fig. 4) has led to much debate as to whether or not such features might characterise glacial terminations in general and further, whether their occurrence reflects spatiotemporal variations in meltwater input to the North Atlantic or a fundamental change in the underlying dynamics of AMOC stability during deglaciation^69–73^. Based on the notion that abrupt oscillations in the AMOC are more pronounced when ice volume is intermediate between full glacial and interglacial conditions (their so-called ‘D-O window’, which is similar to our ‘window of opportunity’), Sima et al.^71^ argued that such oscillations represent a change in the underlying dynamical behaviour of the AMOC and are an intrinsic feature of deglacial transitions which by definition must pass through the D-O window. Indeed, based on an array of evidence^24–27,69^ we are now confident that abrupt mode switches in the AMOC really are a characteristic feature of glacial terminations. However, such a strong feature as the B/A-YD transition (i.e. a return to near glacial conditions following major deglacial warming) seems to be a rare occurrence^25^, such that it is still not clear whether it really reflects a systematic change in AMOC stability or e.g. a chance rerouting of meltwater runoff specific to T1^72,74^. Here we investigate the changing stability of the AMOC with respect to key boundary parameters during deglaciation, and how this might interact with freshwater perturbations to give rise to the various permutations represented by previous terminations.

### A moveable window of AMOC bistability

**Fig. 5:**
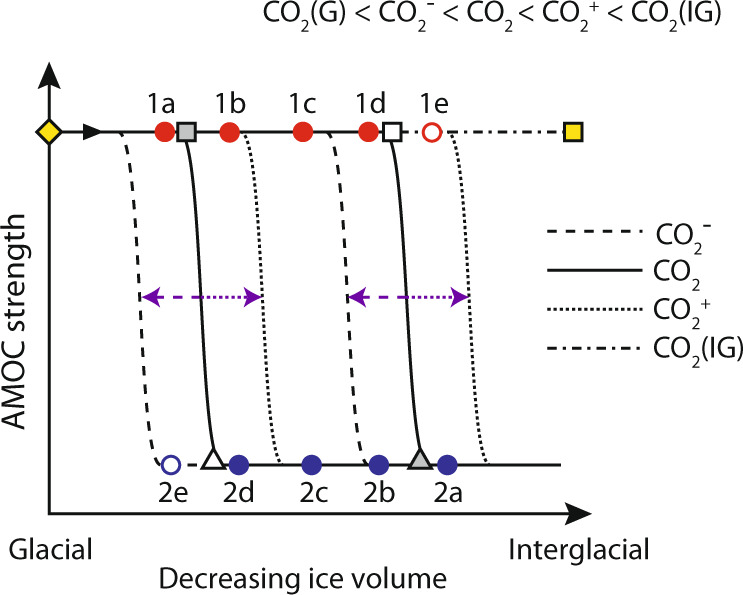
Moveable window of AMOC bistability. Schematic stability diagram for AMOC strength as a function of continental ice volume^57^ for different levels of atmospheric CO_2_^45^ (CO_2 (Glacial)_ < CO_2_^−^ < CO_2_ < CO_2_^+^ < CO_2 (Interglacial)_). Varying atmospheric CO_2_ leads to structural changes in AMOC stability with respect to ice volume (and vice versa) e.g. increasing CO_2_ pushes the window of bistability with respect to ice volume to the right. Yellow diamond represents monostable strong glacial mode of AMOC (a function of large ice sheets) while yellow square represents monostable strong interglacial mode of AMOC (a function of high CO_2_). Other symbols and corresponding letters are referred to in the text.

Many previous studies have pointed to the existence of AMOC bistability with respect to various boundary conditions^45,49,57,75,76^. Moreover, it has been shown that the region of bistability with respect to one parameter can vary, depending on the value of another (Box 1). For example, Zhang et al.^45^ showed that one effect of increasing ice sheet height is to push the window of AMOC bistability with respect to CO_2_ towards lower concentrations of CO_2_. If ice sheets get too large (i.e. similar to glacial maximum conditions) then the region of bistability no longer exists for the observed range of G-IG CO_2_ variability and the AMOC enters a region of monostability. Likewise, for increasing values of CO_2_, the window of bistability with respect to ice sheet height shifts towards smaller and smaller ice sheets until it disappears (monostability) as CO_2_ approaches interglacial values (Fig. 5).

In Fig. 5 we demonstrate how instantaneous transitions in the AMOC could be invoked by structural changes in AMOC stability with respect to one parameter, as another parameter is varied. Beginning in a glacial mode in which the AMOC is monostable strong^45,57^ (solid yellow diamond in Fig. 5) we decrease ice sheet height (while keeping CO_2_ constant) until we cross the grey square to reach 1b. At this point the system is bistable and a transition to a stable weak mode could be invoked by a transient forcing, for example a one-time input of freshwater. As ice sheet height continues to decrease beyond point 1d we leave the window of bistability as the white square is crossed and the AMOC (if still in a strong mode) will experience an abrupt transition to a weak mode (point 2a). All else being equal, ice sheets would then have to grow again by ~18m (SLE)^57^ in order to overcome the inherent hysteresis and transition back into a monostable strong mode (i.e. to cross the white triangle back to point 1a). Alternatively, if CO_2_ were to increase simultaneously (CO_2_^+^ in Fig. 5) then the transition back to monostable strong would occur earlier (i.e. point 2d →1b) as the window of bistability shifts to the right. On the other hand, from bistable strong point 1d an abrupt transition to a monostable weak mode (point 2b) would occur if CO_2_ decreased (CO_2_^−^) even if ice sheets were kept constant.

### Implications for the sweet spot

The considerations above have implications for the occurrence of abrupt shifts in the AMOC within an intermediate climate state and when the AMOC is close to a bifurcation point (which we consider to be more likely within the window of opportunity of intermediate CO_2_ and ice sheet size). As discussed, the region of AMOC bistability with respect to either CO_2_ or ice sheet height is sensitive to changes in the other parameter^45^. This means for example that starting in a weak mode (point 2c in Fig. 5) a weak-strong transition could be triggered by a relatively modest increase in ice sheet height (2c→2d), combined with a modest increase in CO_2_ (shifting the bistable window to the right from ‘CO_2_’ to ‘CO_2_^+^’), resulting in a shift to monostable strong (2d→1b). Likewise, a strong to weak mode transition could be triggered by e.g. the combination of ice decline (1c→1d) plus a CO_2_ decrease (1d →2b). Hence a mixture of ice volume and CO_2_ changes that could come about as a result of millennial-scale changes in the AMOC could themselves increase the likelihood of further abrupt changes in the mode of AMOC and thence further changes in CO_2_ and or ice volume (cf. MIS 3; see previous section; Fig. 3). Such an interpretation could provide a dynamical key to link the sweet spot in the millennial-power band to the local minimum in the distribution histograms of benthic δ^18^O and atmospheric CO_2_ (Fig. 2). In this sense, feedbacks between abrupt AMOC changes and CO_2_ and ice volume might become so effective within the sweet spot that the climate system is forced away from this region as soon as it is entered (Fig. 3).

### Implications for deglaciation

We have already discussed the possibility that exaggerated feedbacks between ocean circulation and atmospheric CO_2_ (and possibly ice volume) might help to amplify the pace of deglaciation as climate passes through the sweet spot for millennial-scale variability. But the notion of a moveable window of AMOC bistability has more fundamental implications for the structure of glacial terminations in general, which we discuss below.

The last deglaciation (T1; Fig. 4) was characterized by a pronounced weakening of AMOC during its earliest phase (Heinrich Stadial 1, HS1)^20^. This event is thought to reflect (at least in part) the addition of freshwater to the surface North Atlantic by decaying ice sheets and is temporally related to the ice rafting event known as Heinrich Event 1 (HE1). Equivalent ice sheet wasting events accompanied every deglaciation of the past 1Myr or so^24,25^, and various studies have attempted to demonstrate that these were also related to a weakening of AMOC^25,26,77^. A large freshwater event could perturb the AMOC into a weak mode even from its glacial monostable strong state but this would only be transient and the AMOC would recover following the end of freshwater input. However, the decrease in ice sheet height that might accompany such a freshwater event could also push the system into a bistable regime or even toward monostable weak (Fig. 5) and in this case the AMOC would remain in a weak mode even after cessation of freshwater input. From this point there are a few possible ways in which a recovery of the AMOC (which must happen at some point during glacial termination) could be achieved:

1. An increase in ice sheet height pushes the system back toward monostable strong (e.g. Fig. 5 point 2d→ 1a). This is unlikely to occur during a deglaciation, when ice sheets are generally thought to be in recession.
2. Recovery within the bistable window (e.g. 2c→1c). This would require an external forcing such as a negative freshwater (salinification) event, which is also unexpected given the tendency for meltwater generation during the deglacial period.
3. Increasing atmospheric CO_2_ pushes the bistable window to the right and forces the system toward monostable strong (e.g. 2d→1b for CO_2_^+^). This would require that the rate of CO_2_ increase effectively outpaced the rate of ice sheet decline, which is possible but probably unlikely again given that ice sheets tend to diminish throughout deglaciation. We suggest that this mechanism could explain AMOC recovery during the Bølling-Allerød.
4. CO_2_ eventually reaches near-interglacial levels and the system is forced into a monostable strong regime. This scenario is inevitable once CO_2_ reaches the desired threshold. The result would be an AMOC recovery towards the end of deglaciation.

We suggest that the last option (4) is the most likely scenario given the reasoning above for options (1) and (2) and the fact that most terminations are not interrupted by a strong oscillation^25^ as we might expect from option (3) (see discussion below).

### Solutions in the time domain

**Fig. 6:**
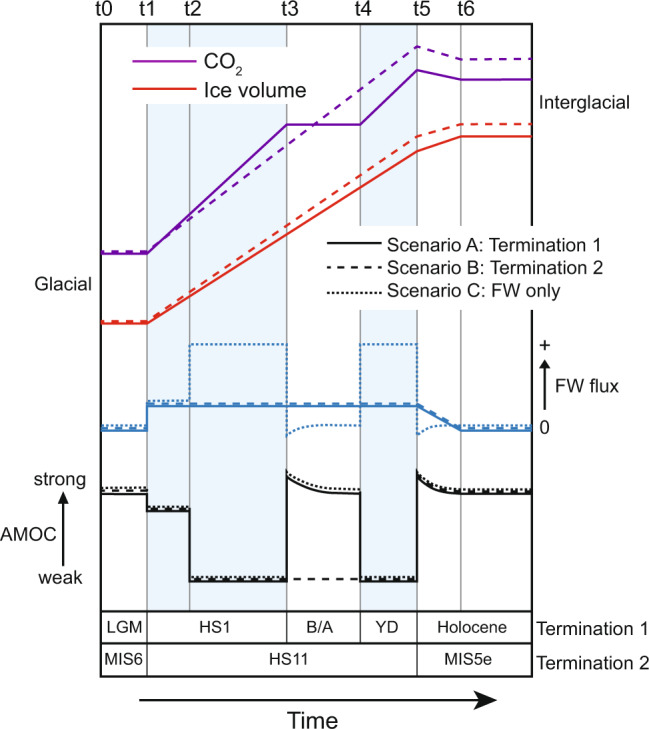
Deglacial changes in structural stability. Idealised timeseries of CO_2_, ice volume and North Atlantic freshwater (FW) flux for last two terminations with corresponding variations in AMOC strength according to text and Fig. 4. Solid curves represent T1; Dashed curves represent T2; Dotted curves represent a scenario for T1 in which variations in the AMOC are forced purely by freshwater (not requiring an underlying structural change in stability). Times t0–t6 are referred to in text.

In Fig. 6 we transfer the stability characteristics from our previous discussion into the time domain for three possible deglacial scenarios (A–C, below). To reflect the decay of ice sheets during deglaciation we make the assumption that a finite flux of freshwater will enter the surface North Atlantic throughout the deglacial period. If the AMOC enters a bistable regime during that time, this flux is sufficient to force it into the weak mode and recovery will not occur unless the system experiences a structural change in stability. It should be stressed that while the bistable regime and deglaciation may overlap in the time domain, they are not synonymous: the bistable regime can exist whenever ice volume and CO_2_ are at intermediate values (as apparent during MIS 3). Furthermore, if enough freshwater is added, this could induce a weak mode even when the AMOC is in a monostable strong regime (while ice sheets are still very large) and this could modulate the precise timing or occurrence of abrupt transitions during deglaciation beyond those predicted by stability changes alone.

Scenario A: The HS1-B/A-YD triptych (Termination 1; solid curves in Fig. 6). Starting in a glacial mode (t0 in Fig. 6; yellow diamond in Fig. 5), warming northern hemisphere summers induce melting of northern continental ice sheets. The resulting freshwater tends to weaken the AMOC (t1) and decreasing ice sheet height pushes it into a bistable regime so that a weak mode becomes inevitable (HS1): t2 in Fig. 6; 1a→2d in Fig. 5. During this period of weakened circulation increasing atmospheric CO_2_ pushes the window of bistability to the right fast enough to outpace the decline in ice sheet height, triggering a recovery of AMOC as the system is forced back toward monostable strong (B/A): t3; 2d→1b (CO_2_^+^). Note that recovery of the AMOC in this scenario can occur even in the presence of continued freshwater input^45^. Within Termination 1, the deglacial rise in atmospheric CO_2_ stagnates during the B/A such that the return to a weak mode of AMOC becomes inevitable as ice sheets continue to decline, pushing the system back towards bistability and possibly to monostable weak (YD): t4; 1b→c2(→2a). Atmospheric CO_2_ then starts to rise again and eventually reaches a level sufficient to induce an interglacial monostable strong mode of AMOC (yellow square in Fig. 5) and a second recovery is achieved (t5; end of YD).

Scenario B: ‘Standard’ termination (e.g. Termination 2; dashed curves in Fig. 6). Starting in a glacial mode (t0 in Fig. 6; yellow diamond in Fig. 5), warming northern hemisphere summers induce melting of northern continental ice sheets. The resulting freshwater tends to weaken the AMOC (t1) and decreasing ice sheet height pushes it into a bistable regime so that a weak mode becomes inevitable (terminal HS event): t2; 1a→2d. As deglaciation proceeds, increasing CO_2_ and wasting ice sheets keep pace with one another so that the AMOC remains in a bistable or monostable weak mode until CO_2_ eventually reaches a level sufficient to induce an interglacial monostable strong mode of AMOC (yellow square) and recovery is achieved (t5). Brief cold events such as the 8.2ka event and the cold event following HS11 recorded at ODP Site 983 (Fig. 4) may be expected as northern ice sheets continue to decay. However, these are distinct from the YD because they occur within an interglacial state (when CO_2_ is high and the AMOC is monostable strong) and are therefore transient by nature i.e. solely driven by e.g. freshwater fluxes.

Scenario C: No change in structural stability of AMOC (dotted curves in Fig. 6). Variations in AMOC are forced purely by changes in freshwater input to the surface North Atlantic.

A test of these predictions requires, at the very least, robust records of atmospheric CO_2_ and ice volume (or preferably ice sheet height and configuration). However, while CO_2_ reconstructions are available back to 800ka, reliable sea-level reconstructions are limited to the last deglaciation (note disagreement among SL reconstructions across T2 in Fig. 4). As we discuss in the final section, this is a critical area for future study. Furthermore, Scenarios A and B could be modified by variable addition of meltwater during deglaciation. For example, AMOC recovery could be delayed beyond entering a monostable strong regime until freshwater addition waned sufficiently. The interplay between freshwater and structural changes in stability might also explain some of the more nuanced features observed during previous terminations (e.g. the 2-part division of HS1^78^ or the partial recovery of AMOC during T3^25^). Of relevance here is a newly published study^5^ in which the authors posit a direct link between insolation forcing and the duration of glacial terminations. Quantifying the duration of a glacial termination using benthic δ^18^O is not ideal (as alluded to in the previous section) but nevertheless the results are instructive since they suggest that higher levels of insolation might lead to a shorter termination by driving faster ice sheet decay. According to our arguments, in these cases we should probably not expect termination to be interrupted by an early recovery of the AMOC (SL rises too fast with respect to CO_2_). Furthermore, by their^5^ calculations Termination 1 is not only relatively long in duration (ranking joint 4th longest of the 11 analysed) but is also longer than might be predicted from its insolation (in this case using integrated summer energy as the relevant quantity). Perhaps its longer duration (i.e. slower SL rise with respect to CO_2_) made Termination 1 particularly prone to an early recovery of the AMOC.

## Early interglacial legacy of deglacial climate instability

So far we have described the interplay between millennial and orbital-timescale variations with respect to glacial terminations but as discussed recently^25^ these interactions are likely to have lasting effects during the period immediately following termination (i.e. during early interglacial times). This becomes important when making comparisons between interglacials whose preceding terminations may have been very different. Such comparisons are important in light of ongoing changes in e.g. greenhouse gas concentrations, which need to be viewed within the context of natural variability to allow robust differentiation of potential human influences on the climate system^79^. For example the rise in atmospheric CH_4_ and CO_2_ over last ~5 and ~8 kyr (respectively) of the Holocene^80,81^ has sparked debate over whether or not humans have been influencing climate for many thousands of years^82–85^.

At the heart of these discussions lies the contrasting histories of both CH_4_ and CO_2_ over the course of the Holocene (which experienced upwards trends in both gases) as compared with previous interglacials (which experienced downward trends in some cases e.g. MIS 7, 9 and 19)^86^. In a recent study^25^ we made the case that the earliest portion (first few kyr) of several previous interglacials should not be included when making these comparisons if quasi-equilibrium is a prerequisite for such comparisons to be valid. We defined (quasi-)equilibrium as a (hypothetical) situation in which Earth’s climate is equilibrated with respect to key boundary conditions including orbital configuration, ice volume and atmospheric CO_2_ concentration and we argued that the earliest parts of many previous interglacials were not at equilibrium thanks to continued adjustment of ocean circulation patterns following deglacial oscillations of the AMOC (note the peculiar coincidence of high amplitude millennial activity and high CO_2_ associated with deglacial periods as mentioned earlier; Fig. 2e ii). We concluded by questioning whether the downward trends in CO_2_ observed for MIS 7, 9 and 19 would remain if the initial period of higher and decreasing CO_2_ associated with AMOC recovery (equivalent to the interval between t5 and t6 in Fig. 6) were omitted from analysis. Below we make the case that the Holocene upward trend in CO_2_ may be considered anomalous a priori thanks to the early recovery of AMOC midway through Termination 1, in which case the early intervention of humans need not be invoked to explain the apparent difference between the Holocene and previous interglacials.

Deaney et al.^23^ compared the evolution of atmospheric CO_2_ across the last two terminations and concluded that the larger apparent magnitude of CO_2_ change across T2 was due to the late recovery of AMOC associated with that termination (e.g. Fig. 4). The continuous rise in CO_2_ associated with Heinrich Stadial 11 (HS11) and subsequent ~20 ppmv jump in CO_2_ associated with recovery of the AMOC ~129 ka resulted in a larger net change in CO_2_ across T2 than the corresponding change across T1. Thus, atmospheric CO_2_ was effectively lower at the onset of the Holocene than it might otherwise have been if the B/A had not occurred (an analogous claim was made recently concerning mean ocean heat content, which was also higher at the onset of MIS 5e than it was at the start of the Holocene^87^). This logic was borne out in a modelling study by Ganopolski and Brovkin^38^ in which they found that a pronounced overshoot in CO_2_ occurs at the beginning of an interglacial when recovery of AMOC happens only at the end of termination. This is followed by a rather constant or even decreasing trend in CO_2_ during the subsequent interglacial. In contrast (and analogous to T1) if a termination is interrupted by an early recovery of the AMOC, CO_2_ will tend to be lower at the start of the subsequent interglacial and proceed to increase throughout the next several thousand years.

We note that CO_2_ also increased throughout MIS 11, which has been used as an analogy for MIS 1 because of the similarity in insolation forcing^88^. Indeed, insolation forcing across T1 was much weaker than e.g. T2 because of lower eccentricity and this has been suggested as a possible reason why an early AMOC recovery did not occur during T2^72^, However, more recently MIS 19 has been advocated as a better insolation analogue for MIS 1^86^ and it is notable that T9 (into MIS 19) did not experience an early AMOC recovery^25^ and CO_2_ decreased throughout MIS 19, following an initial overshoot^25^.

Earlier we made a case that Termination 1 (T1) was unusual in the sense that it was interrupted by an early recovery of the AMOC (associated with the Bølling/Allerød). Given the tendency for changes in ice volume and CO_2_ to coincide during deglaciation, together with the ubiquitous input of freshwater, we argue that the most likely case for a typical termination is Scenario B: essentially an uninterrupted period of weakened AMOC and consequently a late recovery of AMOC (as compared with T1). Following this logic and the studies mentioned above^23,38^ we conclude that the relatively low concentration of CO_2_ observed at the onset of MIS 1 (compared for example with MIS 5, 7 and 9), and its subsequent increase throughout the Holocene, may also be considered as atypical with respect to other interglacials, regardless of any influence that humans may or may not have had. This should be taken into account when making comparisons between the Holocene and more typical interglacials such as MIS 5.

## Summary and outlook

In this perspective we have highlighted progress in our understanding of glacial terminations as the ultimate expression of the interaction between millennial and orbital-timescale variations in Earth’s climate. Specifically we discussed the importance of feedbacks, which appear most accentuated within the sweet spot of millennial activity, and in particular during glacial termination when they help to explain the magnitude and rapidity of these events. We then made the case that structural changes in the stability of the AMOC can explain the occurrence of terminal AMOC oscillations such as the HS1-B/A-YD triptych. Finally we argued that the unusual AMOC oscillation of Termination 1 led to the low initial concentration and subsequent rise of atmospheric CO_2_ throughout the Holocene, which itself may therefore be considered unusual a priori (without the need to call on human intervention).

However, there are still major gaps in our knowledge, which need to be filled before we can test some of the hypotheses put forward here. For example, sea-level reconstructions beyond the last glacial maximum are uncertain and inconsistent (e.g. throughout MIS 3^60,61^ and across MIS 5a/4^89,90^ and Termination 2^91–94^; Fig. 4), making it difficult to test our ideas about changing AMOC stability as a driver for deglacial oscillations in ocean circulation or even to assess the boundary controls on the occurrence of millennial-timescale variability on G-IG timescales. More robust sea-level reconstructions are therefore required that will allow us to compare the relative rates of CO_2_ rise and ice sheet decline during previous terminations and assess our assertions for example about the uniqueness of the B/A-YD oscillation.

An area of gathering momentum is model intercomparison. Many of the exciting theoretical discoveries made over the past few years have been obtained using a single model, leaving their results vulnerable to possible inadequacies in a particular model. Projects such as PMIP^94,95^ will allow more confidence to be gained (or not) in proposed mechanisms as will efforts to compare unforced (e.g. without freshwater forcing) AMOC oscillations in a variety of different model setups. To date most simulations of abrupt climate variability have been forced with freshwater but a growing number of models display forms of ‘unforced’ oscillations (or at least strongly non-linear responses to gradually changing boundary conditions). Given the apparent importance of factors other than freshwater forcing we advocate a community effort to simulate abrupt transitions through other means. Ultimately, the incorporation of an interactive carbon cycle and ice sheet dynamics (including e.g. solid earth and ice shelf cavity processes) will allow a fuller representation of the mechanisms involved in the coupled earth system. In this way we will be able to vastly improve our understanding of how internal climate system dynamics respond to external forcing across deglacial transitions and finally reach a turning point in our understanding of glacial terminations. This knowledge is not merely of academic interest; uncertainties over the location of stability thresholds with respect to our current climate mean that we cannot rule out the possibility that the AMOC is already within a regime of multiple equilibria with respect to North Atlantic freshwater forcing^96^.

## Data Availability

The datasets analysed during the current study are available in the PANGAEA repository, 10.1594/PANGAEA.904398.
